# Editorial: Adaptive immune regulation of non-alcoholic steatohepatitis (NASH)

**DOI:** 10.3389/fimmu.2026.1846400

**Published:** 2026-04-23

**Authors:** Haiguang Wang

**Affiliations:** 1 Department of Integrative Biology and Physiology, University of Minnesota, Minneapolis, MN, United States; 2 Center for Immunology, University of Minnesota, Minneapolis, MN, United States

**Keywords:** B cells, erythroid cells, iNKT (invariant natural killer T cell), MASH - metabolic dysfunction-associated steatohepatitis, myeloid cells, NASH - non-alcoholic steatohepatitis, T cells, Tregs (regulatory t cells)

## Adaptive immunity as a critical regulator of NASH pathogenesis

Metabolic stress induced persistent immune activation drives the progression of metabolic dysfunction-associated steatohepatitis (MASH), previously known as non-alcoholic steatohepatitis (NASH) (1–3). Specifically, stressed hepatocytes can release lipids, cytokines, chemokines, and cell death-associated signals that recruit and activate lymphocytes. These activated lymphocytes then act on macrophages and stromal cells, influencing whether simple steatosis resolves or progresses to fibrosis, cirrhosis or even liver cancer (4, Cebi and Yilmaz). This Research Topic is based on a straightforward premise: In NASH, the adaptive immune system does not merely reflect tissue injury, it can actively shape disease progression. The papers in this Research Topic cover effector cytokine networks, myeloid-regulatory circuits, B cell and T cell modules, and less conventional immunomodulators. Taken together, these articles suggest that adaptive immune responses play a crucial role in determining whether steatosis remains limited or progresses to fibrosis and malignancy.

## T and B lymphocytes in context

The review by Cebi and Yilmaz summarized the adaptive immune landscape of NASH, with an emphasis on lymphocyte trafficking to the liver. It outlines the effector functions of distinct T and B cell subsets, as well as the links between steatohepatitis and hepatocellular carcinoma. Their review points out that immune populations must be interpreted in context. Trafficking patterns, tissue localizations, and effector function together define the role of immune cells in disease.

Furthermore, Abdelnabi et al. focus on interleukin-17 (IL-17) and interleukin-22 (IL-22), showing that cytokine context helps determine whether immune responses worsen hepatic injury or support tissue protection. They suggest that IL-17 is a driver of hepatic inflammation and fibrosis. It not only influences immune cell trafficking, but also directly activates stellate cells. In contrast, IL-22 emerges largely as a tissue-protective cytokine with therapeutic potential, while there are sex-dependent effects.

Li and Xia summarize the role of B cells in NASH and further suggest that B cells are an integral part of the gut-liver axis, encompassing the functions of intestine-derived IgA^+^ B cells, antibodies, and regulatory B cells. Their review highlights the multifaceted role of B cells in NASH pathogenesis, showing that B cells can directly regulate T cell activation and differentiation, macrophage responses, and stellate cell-driven fibrogenesis.

## Myeloid-regulatory circuits that set immune tone

In steatohepatitis, activation of hepatic immune cells unfolds alongside ongoing injury and repair of hepatocytes. Therefore, the handling of dead and dying cells by macrophages is central to the immune response. Sheng et al. review macrophage efferocytosis and emphasize that the metabolites produced during the phagocytic clearance process are immunologically instructive, shaping macrophage reprogramming and downstream immune responses, which may be therapeutically relevant.

Zhao et al. shift the discussion to macrophage-Treg crosstalk in MASLD and emphasize that these interactions change substantially between homeostasis and disease. By comparing the Kupffer cell-Treg interactions in homeostasis with the accumulation of monocyte-derived macrophages as well as the rewiring of cytokine and chemokine networks during disease, they underscore that such regulatory pathways are dynamic. Whether a given pathway restrains inflammation or propels pathology depends on the stage of the disease and tissue environment.

## Noncanonical regulators and lipid-reactive lymphocytes

Immune regulation in NASH also involves cell types not typically centered in adaptive immunity. Niu and Zhang review the immunomodulatory roles of erythroid-lineage cells, highlighting the immature CD71^+^ erythroid cells as suppressors of T cell responses through arginase, reactive oxygen species, PD-L1, TGF-β, and IL-10. Though it remains to be elucidated whether this pathway plays a major role in metabolic liver disease, it broadens the field by linking systemic physiology to immune regulations during chronic liver disease.

Finally, Hopkins et al. present an original research study on invariant natural killer T (iNKT) cells and lipid-associated signals in peanut allergy. Although the study is not liver-specific, iNKT cells are highly enriched in the murine liver. The study also resonates with the biology of steatohepatitis by underscoring that lipids can serve as immune signaling molecules, and that lipid-reactive lymphocytes can be reshaped in both number and phenotype during inflammation.

## Outlook for future research

Collectively, these articles suggest that defining interactions among cells, rather than simply enumerating immune populations, is essential in understanding the immune regulation of steatohepatitis. Several questions remain, however, such as how antigens, cytokines, and tissue niches instruct and reinforce effector or regulatory states? At what point along the path from steatosis to fibrosis and cancer are these states established, and when can they still be reset? Which immune programs can be targeted without compromising host defense? Ultimately, the main challenge is to determine whether these immune mechanisms can be translated into stage-specific biomarkers and/or rational therapeutic strategies.
